# Indirect Health Effects of COVID-19: Unhealthy Lifestyle Behaviors during the Lockdown in the United Arab Emirates

**DOI:** 10.3390/ijerph18041964

**Published:** 2021-02-18

**Authors:** Hadia Radwan, Mahra Al Kitbi, Hayder Hasan, Marwa Al Hilali, Nada Abbas, Rena Hamadeh, Eman Rashid Saif, Farah Naja

**Affiliations:** 1 Department of Clinical Nutrition and Dietetics, Research Institute of Medical & Health Sciences (RIMHS), College of Health Sciences, University of Sharjah, Sharjah 27272, United Arab Emirates; hradwan@sharjah.ac.ae (H.R.); haidarah@sharjah.ac.ae (H.H.); 2 Health Promotion Department, Supreme Council for Family Affairs, Sharjah 27272, United Arab Emirates; mahra@scfa.shj.ae (M.A.K.); eman.Rashed@scfa.shj.ae (E.R.S.); 3 Clinical Nutrition Department, Al Qassimi Hospital-Ministry of Health and Prevention, Sharjah 27272, United Arab Emirates; marwa.alhilali@mohap.gov.ae; 4 Department of Nutrition and Food Sciences, American University of Beirut, Beirut 1107, Lebanon; na280@aub.edu.lb (N.A.); rh213@aub.edu.lb (R.H.)

**Keywords:** COVID-19, diet, lifestyle behaviors, UAE, lockdown

## Abstract

*Background*: Lockdown measures were implemented in many countries to limit the spread of the COVID-19 pandemic. However, such restrictions could precipitate unintended negative consequences on lifestyle behaviors. The main objective of this study was to investigate the prevalence and determinants of unhealthy behavior changes during the COVID-19 lockdown among residents of the United Arab Emirates (UAE). *Methods*: A cross-sectional web-based survey of adults residing in the UAE was carried out during lockdown (n = 2060). Using a multi-component questionnaire, the collected data included questions regarding the following lifestyle changes: Increased dietary intake, increased weight, decreased physical activity, decreased sleep, and increased smoking. An unhealthy lifestyle change score was calculated based on the number of unhealthy lifestyle changes each participant reported. In addition, sociodemographic and living conditions information was collected. Descriptive statistics as well as simple and multiple linear regression analyses were used to examine the prevalence and determinants of the unhealthy lifestyle changes considered in this study. *Results*: Among the unhealthy lifestyle changes examined, increased food intake was the most common (31.8%), followed by decreased physical activity (30%), increased weight (29.4%), decreased sleep (20.8%), and increased smoking (21%). In addition to identifying the correlates of each of the aforementioned lifestyle changes, the results of the multiple regression linear analyses revealed the following correlates for the overall unhealthy lifestyle change score: females (β = 0.32, CI: 0.22; 0.42), living in an apartment (β = 0.12, CI: 0.003; 0.23) and being overweight/obese (β = 0.24, CI: 0.15; 0.32) had higher scores, while older adults (>40 years) had lower scores (β = −0.23, CI: −0.34; −0.12). *Conclusions*: The COVID-19 lockdown has resulted in a high prevalence of unhealthy lifestyle behaviors and practices among UAE residents. The findings of this study provided the evidence base for officials to design interventions targeting high-risk groups and aiming to improve healthy lifestyle factors among residents during the pandemic.

## 1. Introduction

Late in 2019, a new coronavirus called severe acute respiratory syndrome coronavirus 2 (SARS-CoV-2) or coronavirus disease (COVID-19) emerged.It is a highly contagious viral disease, which appeared first in Wuhan, China [1], and then rapidly spread within China and worldwide. This leads the World Health Organization (WHO) on 11 March 2020, to acknowledge COVID-19 as a global pandemic [2]. As of 26 June 2020, there are close to 10 million confirmed cases in 215 countries [3] and the first detected case in the United Arab Emirates (UAE) was on 19 January 2020 [4] with 46,973 confirmed cases as of 26 June 2020 [5].

So far there is no radical treatment for the COVID-19 infection, and the only way to battle this pandemic is by increasing awareness and prevention through the implementation of isolation measures and enhancement of the function of the immune system [6,7]. To combat the COVID-19, many countries have applied preventive measures such as disinfection procedures and partial or complete lockdown to slow the spread of the virus. UAE health authorities are implementing extensive preventative measures to protect public health in line with the World Health Organization’s rules and regulations. On 22 March 2020, the UAE authorities applied both strict infection control and partial lockdown [8], which forced many people to stay at home (study and work from home). Nevertheless, such action may exert a sudden and drastic change in the lifestyle of the population. The lockdown has been postulated to influence lifestyle habits by increasing staying home, excessively storing food, disruption of one’s routine and heightened anxiety hearing the evolving news of the virus and its spread [9]. Such a situation may lead to a change in dietary habits, physical activity, and sleep patterns, in addition to psychological impact [9,10,11,12].

In fact, this new situation may limit access of individuals to daily shopping and affect their choices for a healthy balanced diet with subsequent dependence on highly processed, ready-to-eat cereals and junk foods, which are high in salt, sugar, and fats. Such dietary habits will increase the risk factors of chronic diseases like obesity, heart disease, stroke, type 2 diabetes, some cancers, and chronic kidney disease [13]. Moreover, restricted movement due to lockdown may force many people to stay home and to limit their physical activities [10] and results in more sedentary behavior, which is associated with an increased risk of chronic disease [14]. Noteworthy is the fact that gyms and recreational areas were also included in the lockdown closures and hence were not available for individuals to exercise and carry out leisure physical activity. Furthermore, it has been reported that the lockdown measures could be associated with distressing experience and boredom because the situation is taking the world into uncharted waters. Such circumstances could be triggered by a loss of usual routine and reduced social and physical contact with others [15]. Studies have shown that sleep is crucial for emotional and mental wellbeing and helps to confront stress and anxiety [11]. However, sleep patterns of individuals during pandemic containment could be disturbed.

In the UAE, reports prior to the COVID-19 pandemic, raised alarming concerns regarding a high prevalence of lifestyle related diseases and their risk factor in the country. For instance, 77% of all deaths in the UAE was attributed to cardiovascular disease (CVD), cancer, diabetes, and chronic respiratory disease and the probability of dying prematurely from one of these diseases was estimated at 17% [16]. Among the most common risk factors for these diseases in the UAE were high body mass index, high systolic blood pressure, high fasting plasma glucose, and high total cholesterol [17]. Therefore, it became critical to examine the effect of lockdown on lifestyle habits to develop evidence-based preventions programs aiming to alleviate the heavy burden of unhealthy lifestyle and its consequences on the health of the population in the UAE. Hence, the main objective of this study was to investigate the prevalence and determinants of unhealthy behavior changes during the COVID-19 lockdown among UAE residents, including dietary intake, weight status, physical activity, sleep, and smoking. A secondary objective was to examine the perceptions of healthy practices as well as mental health during the COVID-19 lockdown among study participants.

## 2. Materials and Methods

### 2.1. Study Design and Participants

A cross-sectional web-based online survey was carried out between the 5–18 May 2020 to investigate the health and dietary practices of adults residing in the UAE during the COVID-19 lockdown in the country. The questionnaire link was distributed to 13,500 adults via emails and mobile messages. The list of email addresses and phone numbers was obtained from the database of the Supreme Council for Family Affairs, Health promotion Department, Sharjah and their network in the various Emirates of the UAE. Adults, males and females, aged 18 years and older and residing in the UAE were eligible to complete the questionnaire. Participants ought to be conversant in English or Arabic language. The online survey consisted of a link to an internet-based questionnaire on Google forms with closed-ended questions in English and Arabic. Before starting filling out the online survey, participants were presented with an information form which described the purpose of the study, procedure, time needed to complete the questionnaire as well as the voluntary nature of participation. The informed consent was obtained electronically. It was written in simple and understandable terms on the first page of the questionnaire. Before enrollment, the participant should click ‘Agree’ on the consent form, then he/she can proceed to completing the questionnaire. Otherwise, if the subject did not agree on the consent form, he/she will not be able to proceed and will automatically exit the online link. Ethical approval was obtained from the Research and Ethics Committee at the University of Sharjah (REC-20-05-05-01). The anonymity of the participants was guaranteed during the data collection process.

### 2.2. Questionnaire Used in Data Collection

In order to compile the list of questions to be included in the survey, a triangulated approach was used. First, a thorough review of the literature was conducted in order to identify the main themes that are important in the context of a lockdown. Second, a group of experts, including a nutritionist, a public health worker, and a community activist deliberated on the context specificity of the themes and formulated the questions to be included within each theme. The experts reviewed the questionnaire to ensure its content as well as the face validity. Lastly, the developed questionnaire (originally written in English) was translated into Arabic and then back-translated into English. An expert who is bilingual and whose native language is Arabic translated into and from Arabic. It was then back-translated into English by a bilingual expert panel whose native language is English. Both versions of the questionnaire were pilot-tested for clarity and simplicity and logical flow of the questions. The pilot testing of the questionnaire was undertaken with a convenient sample of adults including UAE nationals as well as adults from other nationalities residing in the UAE. Some questions were removed because of redundancy and some were reworded for clarity.

The final version of the questionnaire consisted of 30 questions grouped into four main sections: (1) Sociodemographic characteristics, (2) Dietary habits, (3) Lifestyle practices, and (4) Healthy practices during the COVID-19. The first part included questions related to the socio-demographic profile of the participants (age, gender, marital status, educational level, region, type of residence, no of children, etc.) and the weight (kg) and height (cm) of the participants. Both weight and height were self-reported by the participants. The body mass index (BMI) was calculated by dividing the weight (in kg) by the height (in m^2^). Participants were then classified into four categories, underweight (<18.5 kg/m^2^), normal (18.5–24.9 kg/m^2^), overweight (25.0–29.9 kg/m^2^), and obese (≥30.0 kg/m^2^) (WHO, 2004). The second part of the questionnaire included questions related to dietary habits. The focus was on the quantity and quality of the diet, such as the amount of food consumed during lockdown (decrease, same or increased), the type of foods that were mostly consumed during the lockdown, and which foods’ intake increased during the lockdown. The section addressing the lifestyle included questions related to weight changes (decreased, same, increased), physical activity (decreased, same, increased); smoking (decreased, same, increased) and the number of hours of sleep per day (decreased, same, increased). With regards to the healthy practices during COVID-19, participants were asked if they wore masks and gloves when going out, the frequency of grocery shopping, and if they disinfected and sterilized the food items purchased. The questionnaire required approximately 10 min to complete. The questionnaire was included in this manuscript as Supplementary Materials.

### 2.3. Statistical Analysis

Data analysis was conducted using IBM SPSS (IBM Corp. Released 2017. IBM SPSS Statistics for Windows, Version 25.0. Armonk, NY: IBM Corp.). Descriptive statistics were presented as frequency and percentage. The main outcomes were unhealthy dietary and lifestyle changes described as decreased physical activity (vs. same or increased), decreased sleep (vs. same or increased), increased smoking (vs. same or increased and non-smokers), increased weight (vs. same or decreased) and increased food intake (vs. same or decreased). An unhealthy dietary and lifestyle changes score was generated whereby participants will receive 0 to 5 points based on the number of unhealthy dietary and lifestyle changes they presented. Each main outcome counted as 1 point towards the overall score. The total score was additive, with each outcome having equal weight to the score. As such, a higher score indicated a higher adoption of unhealthy lifestyle during the lockdown. Simple and multiple logistic regressions were conducted to describe the associations between each of the aforementioned outcomes (decreased physical activity, decreased sleep, increased smoking, increased weight and increased food intake), and sociodemographic characteristics of the study population. Simple and multiple linear regression analysis were used for the association between the unhealthy dietary and lifestyle changes score and sociodemographic characteristics. In the adjusted models, variables with a significant *p*-value (<0.05) at the univariate level were entered in the model. Results of the logistic and linear regression models were expressed, respectively, as Odds Ratios (OR) and β coefficient with 95% Confidence Intervals (CI). *p*-values less than 0.05 were considered statistically significant.

## 3. Results

Out of 13,500 sent links, 2135 responses were received. Of those, 2060 were returned with complete answers, resulting in a response rate of 15.8%. Table 1 described the sociodemographic characteristics of the study population. Three-quarters (75.1%) were females, 31.7% were between 18 and 30 years old, 38.4% were between 31 and 40 years old, and 29.9% were older than 40 years. Over half of the study population (63.8%) were married and the majority had a university degree (76%). One in two participants was an Emirati (50.7%), the rest of the sample was distributed as follows: Arabs (33.4%), Asians (12.4%), Western (2.4%), and other nationalities (1.1%). The most commonplace of residence was Sharjah (68%) followed by Dubai (11.8%), Ajman (8.8%), Abu Dhabi (5.8%), Ras Al Khaima (RAK) (2.6%), Umm Al Quwain (UAQ) (1.6%) and Fujairah (1.4%). The distribution of the study sample across the various emirates was illustrated on the map of the UAE in Appendix A. Almost two-thirds (60.7%) reported living in a house with a garden or yard. One in four (25.5%) were not working at the time of completing the questionnaire and 54.8% were working from home. Among the study participants, 38.4% had no children, 30% had one or two children and 31.7% had three or more. In terms of BMI status, 64.7% of the study population were either overweight or obese (≥BMI of 25 kg/m^2^) (Table 1).

The description of the lifestyle behaviors as well as the overall unhealthy lifestyle score is presented in Table 2. For all the lifestyle characteristics considered, almost 50% of the study population did not change behavior (physical activity (53.5%); food intake (51.5%), weight status (47.5%), smoking (40%), and sleep (53.9%)). As for the overall unhealthy lifestyle score, 30% of participants had a score of 0, indicating no unhealthy change in any of the behaviors. 62.1% had 1 or 2 unhealthy behaviors and 9% had 3 or more unhealthy behaviors.

**Figure 1:**
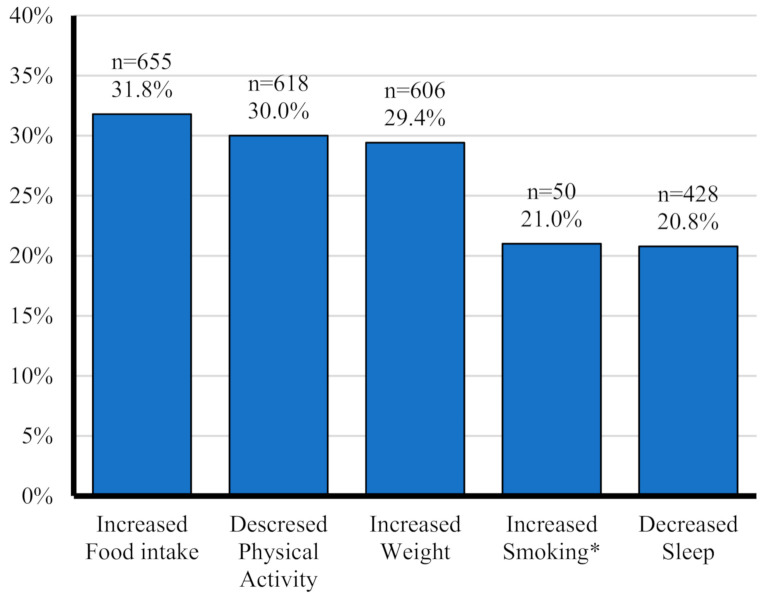
Prevalence of unhealthy lifestyle behaviors among study participants (*n* = 2060). * Percentage among smokers (N = 238).

The prevalence of each of the five unhealthy behaviors considered in this study is illustrated in Figure 1. Increased food intake was the most common, reported by 31.8%, followed by decreased physical activity (30%), and increased weight (29.4%). Decreased sleep and increased smoking were reported by 20.8% and 21%, respectively.

The associations of each of these unhealthy lifestyle changes with sociodemographic characteristic, as examined by simple and multiple logistic regression are summarized in Appendix B. Regarding increased food intakes, female sex, being Non-Emirati Arabic (vs. Emirati), having 1–2 children (vs. none), and being overweight or obese were risk factors for increased food intake (females: OR: 1.87, CI: 1.47–2.39; being an Arab: OR: 1.35, CI: 1.02–1.79; having 1–2 children: OR: 1.33, CI: 1.05–1.68; Being overweight or obese: OR: 1.47, CI: 1.19–1.82). On the other hand, being older than 40 years (vs. 18–30 years old) was associated with a lower odd of increasing food intake (OR: 0.57, CI: 0.43–0.86). As for increased weight, the following characteristics were associated with higher odds: females (OR: 1.89, CI: 1.47–2.41), Non-Emirati Arabic (OR: 1.62, CI: 1.22–2.17), Western (OR: 2.06, CI: 1.08–3.93), Asian nationalities (OR: 1.70, CI: 1.15–2.50), living in an apartment or house with no garden or yard (OR: 1.51, CI: 1.15–1.98), and being overweight or obese (OR: 2.25, CI: 1.79–2.83). Decreased physical activity was significantly associated with age, nationality, working from home and BMI: older adults aged more than 40 years (O: 1.61, CI: 1.25–2.08), working from home (OR: 0.7, CI: 0.55–0.89) and overweight or obese (adj OR: 1.38, CI: 1.13–1.72). As for increased smoking behavior, females and older adults (>40 years old vs. 18–30 years old) were less likely to increase their smoking behavior during the lockdown (for females ORs 0.27, CI 0.15–0.49; for older adults: OR: 0.25, CI: 0.11–0.57). In terms of sleeping behavior, females were more likely to experience decreased sleep compared to males, whereas older adults (>40 years old vs. 18–30 years old) were less likely to experience decreased sleep during the lockdown. (Appendix A).

The association of the unhealthy lifestyle and dietary behaviors score with sociodemographic variables are shown in Table 3. The results of the multiple regression analyses showed that females were more likely to have a higher score (Coef. 0.32, CI: 0.22; 0.42) as well as those living in an apartment or a house with no garden or yard (vs. in a house with a garden or yard) (Coef. 0.12, CI: 0.003; 0.23) and those whose BMI ≥ 25 kg/m^2^ (Coef. 0.24, CI: 0.15; 0.32). On the other hand, older adults (>40 years old) were more likely to have a lower score compared to 18 to 30 years old adults (Coef. −0.23, CI: −0.34; −0.12). (Table 3). The reference categories which were used for the various sociodemographic variables in these analyses are indicated in Table 3.

Perception, health, and dietary practices and perceived mental health status related to COVID-19 among the study population were described in Table 4. When asked about their agreement on “Quarantine is a good way to protect my family and to stop the spread of infectious disease outbreaks”, 98.4% of the participants agreed. When going out, 83% reported using both gloves and masks, 14.7% used masks only, 1.3% gloves only, and 1.1% haven’t used any. With regards to dietary practices, a vast majority (90.8%) reported home-cooked food as one of the most consumed food items during the COVID-19 pandemic. The next most reported items were fruits and vegetables (60.2%, each), herbal tea (37%), nuts (34.3%), and sweets (32.6%). As for the type of food the participants consumed more during the COVID-19 pandemic, 84.4% chose cooked food while 21.3% chose salty snacks, 7.1% sweet sacks and only 5.7% chose nothing. The proportion of going grocery shopping increased by 4% of the study population, remained the same for 22.1, and decreased for 22.9% while 51% reported no definite number of times of going grocery shopping. Almost half (48%) of the participants reported ordering food or groceries for delivery and 79.4% sterilize food items when delivered. The most common ways reported to disinfect purchased fruits and vegetables were water only (43.8%), water and vinegar (21.9%) and water, vinegar, and salt (9.8%) whereas 1.8% reported not disinfecting purchased fruits and vegetables. The participants were asked if they felt angrier and stressed during lockdown, 41.8% reported that they felt angrier and 67.2% were more stressed during lockdown. When asked what they did to relieve stress, the majority reported they prayed (33%) or watched movies/listened to music/social media (27%) to relieve their stress. While the rest either ate more (10.5%), some exercised (10.2%), or meditated (10.1%) to relieve stress.

## 4. Discussion

The findings of this study showed that the most prevalent unhealthy change in behavior among participants was increased food intake (32%). The confinement to one’s home and the interruption of the work-related routine could lead to irregular eating patterns and frequent snacking, both of which are associated with increased food intake and consequently a more positive caloric balance [18]. Such an increase in food intake could also be driven by fear and anxiety. During the quarantine, continuously hearing or listening about the pandemic spread and its associated mortality can be stressful [19]. In fact, in this study more half of the participants reported anger and heightened levels of stress during the lockdown. For many people, the response to stress, distress, and emotional disturbance is not to avoid food but possibly to increase the consumption of energy-dense foods [20]. It has been proposed that this emotionally-induced eating comes as a result of interference of eating by emotions, a by-product of emotions, and a consequence of regulatory processes (i.e., emotions may regulate eating, and eating may regulate emotions) [21]. Of concern is the finding of this study that salty and sweet snacks (chips, crackers, cupcakes, cookies) were reported among the foods in which intake has increased most during lockdown. These foods are usually calorie-dense and also high in salt, sugar, saturated fats [22,23]. In addition, the higher consumption of these foods could displace more nutritious options in the diet such as fruits and vegetables. During the COVID-19 pandemic, optimal nutrition intake is paramount given its significant effects on the immune system. In fact, adequate dietary nutrient intakes are postulated to directly affect immunity through gene expression, cell activation, and signaling molecule modifications. In addition, many nutrients are implicated in the proper functioning of the gut microbiota and its composition and hence indirectly modulating the immune response [24]. Therefore, promoting the adoption and maintenance of a healthy and well-balanced diet seems to be a prerequisite for a strong and well-functioning immune system that could withstand the assault of infections, including that of the Coronavirus.

In this study, one in three participants reported lower physical activity levels during lockdown. This significant prevalence of lower physical activity was also reported by a cross-sectional survey among Lebanese adults, whereby 41% reported no physical activity or exercise during lockdown [25]. In fact, staying home for a prolonged period of time may lead to more prevalent sedentary behavior such as excessive sitting/reclining, increased screen time activities (video games, TV watching, use of mobile devices) [26]. The negative consequences of the decrease in physical activity observed during COVID-19 affect both the physical and mental health. On one hand, lower physical activity levels lead to lower energy expenditure, a higher risk of overweight, obesity, and hence worsening of chronic health conditions [27]. In addition, lower levels of exercise have been shown to lower the immune competency and regulation and increase the risk of developing systemic inflammation [28]. Furthermore, accumulating evidence indicated that regular physical activity is beneficial for mental health as it reduces anxiety, depression, and negative mood, and improves self-esteem and improves the overall quality of life [29]. These negative effects of lower physical activity on health, including a higher risk of chronic diseases, lower immunity, and compromised mental health, were in fact implicated in more severe symptoms and worse outcome for COVID-19 [27,30,31]. Therefore, it is recommended to continue to maintain a healthy level of physical activity in a safe home environment using simple and easily implementable exercises such as walking in the house, lifting and carrying objects such groceries, alternating leg lunges, climbing the stairs, stand-to-sit and sit-to-stand using a chair and from the floor, and sit-ups and pushups. In addition, recently there has been an abundance of eHealth and exercise videos, which focuses on encouraging and delivering physical activity through the Internet, mobile technologies, and television [32].

The third most common unhealthy lifestyle behavior among study participants, after the increase in food intake and decreased physical activity was ‘increased weight’. It is, in fact, conceivable that these three unhealthy behaviors are interconnected, whereby the higher intake of food coupled to lower levels of physical activity would lead to a positive energy balance, a weight gain, and higher risk of overweight and obesity. Increasing evidence indicated that obesity is strongly associated with severe symptoms and death from COVID-19 [33,34,35]. For instance, the risk for invasive mechanical ventilation in patients with COVID-19 infection admitted to the Intensive Treatment Unit increased by seven-fold for those with BMI > 35 compared with BMI < 25 kg/m^2^ [33]. In their review of the potential mechanisms by which obesity affects the disease outcome, Sattar et al., suggested that obesity and ectopic fat may lower the cardio-respiratory fitness, increase the cardiovascular susceptibility to immune driven vascular and thrombotic effects, impaired metabolic response, dysregulate the immune response and increase viral exposure, through more viral shedding and viral load in the breath [36]. The higher risk of overweight and obesity during COVID-19 observed in this study is rather alarming when considered in the context of the UAE. Recent population-wide data (before the pandemic) showed that over 65% of adults residing in the UAE were already either overweight or obese, with 57% having central obesity [37]. This high prevalence of obesity is accompanied by a heavy burden of Non-communicable diseases (NCDs) in the country, where the four main NCDs accounted for 77% of all deaths in UAE and 17% of premature deaths (30–70 years) in the year 2019 [17]. Therefore, public health intervention tackling the prevention and management of obesity in the country is needed, now more than ever, in order to not only prevent chronic diseases but also limit the adverse reactions to viral pandemics. Decreased sleep and increased smoking were also among the unhealthy lifestyle changes which were reported in this study. Similar to these findings, previous studies showed that, among individuals who were self-isolated during the COVID-19 outbreak, a worsening of sleep duration and quality was observed [38]. Furthermore, a survey of lifestyle habits conducted in Poland showed that over 45% of smokers experienced a rise in smoking frequency during the quarantine [39]. Both decreased sleep and increased smoking can potentially increase vulnerability to infection by the coronavirus and worsen the clinical symptoms of COVID-19. Sleep is an important modulator of the immune response, whereby shorter duration of sleep was associated with a weaker immunity and an increased susceptibility to infection [40]. Suggested mechanisms for this association included impaired mitogenic proliferation of lymphocytes, decreased HLA-DR expression, the upregulation of CD14+, and variations in CD4+ and CD8+ T lymphocytes [40]. As for smoking, a review of its association with immunity indicated significant impacts of smoking on both innate and adaptive immunity by either exacerbation of pathogenic immune responses or attenuation of defensive immunity [41].

The unhealthy lifestyle changes described in this study call for evidence-based interventions to limit the undesirable effect of lockdown on the health of adults in the UAE, especially among population groups that exhibit a higher likelihood to adopt these unhealthy changes. To that effect, the findings of this study showed that females compared to males were more likely to report increased dietary intake, increased weight, lower physical activity, and decreased sleep. The vulnerability of women to such changes in their lifestyle could be explained by the fact that women, in general, are more susceptible to emotional exhaustion, stress, and their negative health implications [42]. In this study, participants living in an apartment with no yard or garden were more likely to report all of the unhealthy lifestyle changes, except for decreased sleep. This finding underscores the role of built environment in affecting the lifestyle of individuals. A growing body of evidence supports built environment as a health promoter of individuals and communities by influencing risk factors, such as dietary choice and physical activity [43]. In addition to gender and living conditions, weight status was shown to affect the odds of adopting unhealthy behaviors, whereby in this study, having a BMI ≥ 25 kg/m^2^ were more likely to report higher dietary intake, increase in weight, lower physical activity and increased smoking. It has been suggested that during quarantine, patients suffering from obesity may experience higher levels of stress which in turn makes them more vulnerable to over-eating and sedentary lifestyle, thus predisposing them to further weight gain [20]. These findings suggest that obese subjects must be carefully informed about the risk of an unhealthy lifestyle during the quarantine due to the increasing risk of disease associated with obesity. In addition, it is important for overweight and obese subjects to carefully monitor their health and to receive strong psychological support to reduce their stress and anxiety levels.

In this study, among the factors that were associated with lower odds of adopting unhealthy lifestyle changes was older age (>40 years). While older adults were at a higher risk of complications and severity of the coronavirus infection [44,45,46], it appeared that younger individuals seemed to be more vulnerable to the effect of confinement and exhibited an overall higher frequency of unhealthy changes in their lifestyles. It is possible that younger adults are less prepared to cope with the lockdown and its consequences as well as stress, anxiety, and fear.

The findings of this study ought to be considered in view of a few limitations. First, the online nature of the survey limits the ability to objectively verify the data. As such all the changes in the lifestyle are based on self-reports and could have been subject to self-selection and reporting bias. Of concern, is the self-reported nature of weight, height and consequently BMI. It has been suggested that anthropometric measures are subject to systematic reporting biases causing discrepancies between self-reported and measured height and weight [47]. However, given the lockdown situation in the UAE, face-to-face data collection was not possible. Furthermore, a recent study suggested that BMI derived from self-reported weight and height could serve as a potentially valid measure among adult across different socio-demographic groups [48]. Second, the low response rate in this survey could have resulted in a non-response bias and hence limited the generalizability of the findings. Such low response rate of the online survey has been previously reported. A study comparing the response rates between paper and online surveys in a general population health survey showed that the paper mode has significantly higher response as compared to web-based format (OR: 2.04; 95%CI: 1.61–2.59) [49]. Previous literature suggested that the degree of survey fatigue in a society, the social cohesion, and the public attitudes towards the survey industry are among the factors that could lead to low response rate [50]. In the context of this study, survey fatigue could have been a main factor contributing to the relatively low response rate, especially that many research initiatives were launched simultaneously to survey individuals during lockdown in the UAE. However, in the case of this study, the advantages of this method (speed, cost, no physical contact) outweighed its disadvantages and potential biases. Third, in this study, females were more likely to participate in the study (75%) as compared to males (25%). A couple of reasons could have led to this difference. First, during the lockdown, a few jobs, with majority of males, were exempted from compliance with the confinement measures, such as the army, police forces, food service personnel and medical staff. Therefore, females were more likely to observe the lockdown and hence participate to the objectives of this research. Second, male sex has been repeatedly cited as a key factor associated with non-response [51,52], with women presenting higher participation rates in health-related surveys. For instance, the health examination survey participation rates across seven European countries as well as those stemming from the National Health and Nutrition Survey (NHANES) in the USA showed higher prevalence of women as compared to men [53,54]. Fourth, it is important to note that the questionnaire that was used in this study was not validated in the UAE population. That said, for the research question of this study in particular, and given the exceptional and unusual circumstances of the COVID 19 pandemic, there existed no previously validated questionnaire which could serve to examine the lifestyle changes during lockdown in the UAE. A few measures, however, were undertaken in order to improve the validity (content and face validity) and parallel form reliability of the questionnaire including (1) a thorough review by experts in the field of lifestyle and public health, (2) conducting a pilot test assessment on a convenient sample of UAE nationals and adults of diverse nationalities residing in the UAE and (3) translation and back translation by bilingual experts. Lastly, the cross-sectional nature of the study design does not allow inference of causality. Therefore, future research aiming to inform policy and decision makers ought to use more sophisticated designs and analyses such as the Decision Tee, among others.

## 5. Conclusions

Since the outbreak of the COVID-19 pandemic, restrictive measures of confinement and lockdown were imposed in many countries around the world, including the UAE. Although efficient in limiting the spread of the virus, such measures could have detrimental consequences on the lifestyle and wellbeing of individuals. Among potential unhealthy lifestyle changes that could propagate during lockdown are increased dietary intake, increased weight, decreased physical activity, increased smoking, and decreased sleep. The findings of this study highlighted that over half of adults residing in the UAE had one or two of the aforementioned unhealthy lifestyle changes during the lockdown with 9% reporting more than 3. The female gender, living in an apartment and being overweight or obese were more likely to report unhealthy lifestyle changes. In addition, almost half of the study population were angry and felt more stressed during the lockdown. Such findings are important to consider in a country such as UAE, given its high burden of diseases related to unhealthy lifestyle, namely the NCDs. Residents in the UAE should be encouraged to improve their lifestyle to lessen the risk both in the current and subsequent waves of COVID-19. Concerted efforts are needed to develop interventions aiming to alleviate the impact of lockdown on diet and lifestyle. Promoting a healthy diet, regular at-home exercises, avoiding smoking, and working on mental health are key factors to be considered to improve in boosting the immune system. Using the e-platforms for the design and delivery of these interventions to help people adopt sustainable changes could constitute an opportunity for which future studies are needed to determine feasibility and efficacy. Future research is needed to confirm the findings of this study and examine the role of socioeconomic status on changes in lifestyle habits. In addition, follow up studies would be important to shed light on long term impact of the COVID -19 lockdown on lifestyle and health related behaviors of individuals.

## Figures and Tables

**Table 1:** Sociodemographic characteristics of the study population (*n* = 2060).

		*n*	%
Gender	Male	512	24.9
	Female	1548	75.1
Age (years)	18–30	654	31.7
	31–40	791	38.4
	>40	615	29.9
Marital status	Single	639	31.0
	Married	1315	63.8
	Ever married	106	5.1
Education	Up to high school & technical diploma	494	24.0
	University	1566	76.0
Nationality	Emirati	1044	50.7
	Arabic	688	33.4
	Western	49	2.4
	Asian	256	12.4
	Other	22	1.1
Emirates of residence	Sharjah	1401	68.0
	Dubai	243	11.8
	Abu Dhabi	119	5.8
	Ras El Khaimah (RAK)	54	2.6
	Fujairah	29	1.4
	UAQ	32	1.6
	Ajman	182	8.8
Type of living	In a house with a garden or yard	1250	60.7
	In an apartment/a house with no garden or yard	810	39.3
Working status	Not working	525	25.5
	working from home	1129	54.8
	working	406	19.7
Number of children	None	791	38.4
	1–2	617	30.0
	≥3	652	31.7
BMI (kg/m^2^)	Normal (<25)	728	35.3
	Overweight and/or obese (>or = 25)	1332	64.7

Abbreviations: BMI: Body Mass Index.

**Table 2:** Description of dietary and lifestyle behaviors changes during COVID-19 pandemic among the study population (*n* = 2060).

		*n*	%
Physical activity	Increase	340	16.5
	Decrease ^b^	618	30.0
	Same	1102	53.5
Food intake	Increase ^b^	655	31.8
	Decrease	344	16.7
	Same	1061	51.5
Weight	Increase ^b^	606	29.4
	Decrease	476	23.1
	Same	978	47.5
Smoking ^a^	Increase ^b^	50	21.0
	Decrease	93	39.1
	Same	95	39.9
Sleep	Increase	522	25.3
	Decrease ^b^	428	20.8
	Same	1110	53.9
Unhealthy lifestyle score (Mean ± SD: 1.14 ± 0.96)	0	595	28.9
	1–2	1279	62.1
	≥3	186	9.0

^a^ percentage among smokers (N = 238) ^b^ considered as unhealthy behaviors which were added to determine the score.

**Table 3:** Simple and multiple linear regressions describing the association between unhealthy lifestyle score and sociodemographic characteristics in the study population (*n* = 2060).

	Unhealthy Lifestyle Score (*n* = 2060)					
	Model 1			Model 2		
	Crude Coef.	95% CI	*p*-Value	Adj Coef.	95% CI	*p*-Value
**Sex (reference: males)**						
Female	**0.26**	**(0.16; 0.35)**	<0.001	**0.32**	**(0.22; 0.42)**	<0.001
**Age, years (reference: 18–30)**						
31–40	−0.09	(−0.19; 0.01)	0.07	0.11	(−0.21; −0.01)	0.032
> 40	**−0.19**	**(−0.3; −0.09)**	<0.001	**0.23**	**(−0.34; −0.12)**	<0.011
**Marital status (reference: Single)**						
Married	−0.06	(−0.15; 0.03)	0.213			
Ever married	−0.07	(−0.27; 0.13)	0.478			
**Education (reference: up to high school)**						
University	−0.07	(−0.16; 0.03)	0.183			
**Nationality (reference: Emirati)**						
Arabic	**0.1**	**(0.003; 0.19)**	0.042	0.08	(−0.04; 0.2)	0.215
Western	0.1	(−0.17; 0.38)	0.473	0.12	(−0.16; 0.4)	0.395
Asian	0.03	(−0.1; 0.16)	0.619	0.03	(−0.12; 0.19)	0.673
Other	0.21	(−0.19; 0.62)	0.299	0.14	(−0.27; 0.54)	0.507
**Type of living (reference: house with a garden or yard)**						
An apartment/house with no garden or yard	**0.12**	**(0.04; 0.21)**	0.005	**0.12**	**(0.003; 0.23)**	0.045
**Working Status (reference: Not working)**						
Working from home	**−0.1**	**(−0.2; −0.01)**	0.039	0.04	(−0.14; 0.06)	0.436
Working	−0.02	(−0.15; 0.1)	0.741	0.09	(−0.04; 0.21)	0.183
**Number of Children (reference: 0)**						
1–2	0.08	(−0.03; 0.18)	0.144			
>3	−0.02	(−0.12; 0.08)	0.649			
**BMI (kg/m^2^) (reference: <25)**						
≥25	**0.15**	**(0.06; 0.24)**	0.001	**0.24**	**(0.15; 0.32)**	<0.001

Abbreviations: BMI: Body Mass Index, Coef.: Coefficient, adj coef: adjusted coefficient, CI: Confidence interval. Model 1: Crude associations; Model 2: Adjusted for sex, age, nationality, type of living, working status and BMI. Numbers in bold are statistically significant (*p*-value < 0.05).

**Table 4:** Description of, perception, health and dietary practices, and perceived mental health status related to COVID-19 among the study population (*n* = 2060).

		*n*	%
Health practices			
Agreement on “Quarantine is a good way to protect my family and to stop the spread of infectious disease outbreaks”	Agree	2028	98.4
	Disagree	32	1.6
Gloves and Mask use when going out	Gloves	26	1.3
	Mask	303	14.7
	Both	1709	83
	None	22	1.1
**Dietary practices**			
Food items consumed the most during COVID-19 pandemic	Home cooked food	1871	90.8
	Vegetables	1240	60.2
	Fruits	1240	60.2
	Herbal tea	762	37.0
	Nuts	707	34.3
	Sweets	672	32.6
	Cakes and pastries	548	26.6
	Chips and crackers	485	23.5
	Fried food	379	18.4
	Delivery food	127	6.2
	Fast food	92	4.5
	None	85	4.1
Type of food the participants’ consumed more of during COVID-19 pandemic	Cooked food	1739	84.4
	Salty Snacks: chips, popcorn, crackers etc.	439	21.3
	Sweet snacks: Cupcakes, cookies, cakes, etc.	147	7.1
	Nothing/Other	117	5.7
Number of times went grocery shopping per week during COVID-19 lockdown as compared to before COVID-19 lockdown	Increase	83	4.0
	Decrease	471	22.9
	Same	456	22.1
	No definite number of times	1050	51.0
Dietary Ways to disinfect purchased fruits and vegetables	water	902	43.8
	Water + salt	107	5.2
	Water + Vinegar	451	21.9
	Water + Vinegar + salt	201	9.8
	Water + Detergents (Dishwashing liquid)	176	8.5
	Disinfectants for vegetables and fruits	186	9.0
	None	37	1.8
Dietary Ordering food or groceries for delivery	Yes	989	48.0
	No	1071	52.0
Dietary Sterilizing food items when delivered	Yes	1636	79.4
	No	424	20.6
**Perceived mental health status and practices**			
Feeling angry more during the lockdown	Yes	860	41.7
	No	1200	58.3
Feeling more stressed during lockdown	Yes	1385	67.2
	No	1200	58.3
Practices to relieve stress during lockdown	Pray	679	33.0
	Watch movies/music/social media	554	26.9
	Eat more	216	10.5
	Exercise more	209	10.2
	Meditate	210	10.1
	Other (reading, talking to friend, etc.)	129	6.3

**Figure A1:**
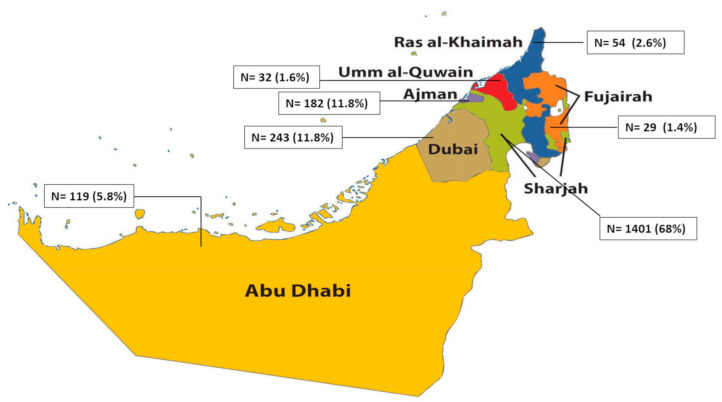
Illustration representing the distribution of the study sample across the map of the UAE.

## Data Availability

The datasets used and/or analyzed during the current study are available from the corresponding author on reasonable request.

## Supplementary Materials

The following are available online at https://www.mdpi.com/1660-4601/18/4/1964/s1.

## Appendix B

**Table A1:** Simple and multiple logistic regressions describing the association between increased food intake and sociodemographic characteristics in the study population (*n* = 2060).

	Increased Food Intake (*n* = 655)						
	Model 1			Model 2		
	Crude OR	95% CI	*p*-Value	Adj OR	95% CI	*p*-Value
Sex (ref: males)						
Female	1.68	(1.34; 2.11)	<0.001	1.87	(1.47; 2.39)	<0.001
Age (ref 18–30)						
31–40	0.95	(0.76; 1.18)	0.63	0.86	(0.67; 1.09)	0.215
>40	0.68	(0.53; 0.86)	0.002	0.57	(0.43; 0.76)	<0.001
Marital status (ref: Single)						
Married	0.95	(0.78; 1.17)	0.635			
Ever married	1.07	(0.69; 1.66)	0.75			
Education (ref: up to high school)						
University	1.13	(0.91; 1.41)	0.265			
Nationality (ref: Emirati)						
Arabic	1.24	(1.01; 1.53)	0.037	1.35	(1.02; 1.79)	0.036
Western	1.37	(0.76; 2.49)	0.295	1.5	(0.78; 2.87)	0.224
Asian	1.16	(0.86; 1.55)	0.331	1.3	(0.89; 1.91)	0.172
Other	0.89	(0.34; 2.29)	0.806	0.83	(0.31; 2.23)	0.716
Type of living (ref: house with a garden or yard)						
An apartment/house with no garden or yard	1.22	(1.01; 1.48)	0.038	1.16	(0.88; 1.52)	0.288
Working Status (ref: Not working)						
Working from home	0.9	(0.72; 1.13)	0.799			
Working	0.93	(0.71; 1.23)	0.381			
Number of Children (ref: 0)						
1–2	1.28	(1.02; 1.6)	0.033	1.33	(1.05; 1.68)	0.02
≥3	1.03	(0.83; 1.3)	0.769	1.25	(0.95; 1.64)	0.116
BMI (kg/m^2^) (ref: <25)						
≥25	1.26	(1.04; 1.54)	0.02	1.47	(1.19; 1.82)	<0.001

Abbreviations: BMI: Body Mass Index, Coef.: Coefficient, adj coef: adjusted coefficient, CI: Confidence interval. Model 1: Crude associations; Model 2: Adjusted for sex, age, nationality, type of living, number of children, and BMI.

**Table A2:** Simple and multiple logistic regressions describing the association between increased weight and sociodemographic characteristics in the study population (*n* = 2060).

	Increased Weight (*n* = 606)					
	Model 1			Model 2		
	Crude OR	95% CI	*p*-Value	Adj OR	95% CI	*p*-Value
Sex (ref: males)						
Female	1.41	(1.13; 1.78)	0.003	1.89	(1.47; 2.41)	<0.001
Age (ref 18–30)						
31–40	0.76	(0.61; 0.95)	0.015	0.69	(0.53; 0.89)	0.004
>40	0.66	(0.52; 0.85)	0.001	0.55	(0.41; 0.73)	<0.001
Marital status (ref: Single)						
Married	0.91	(0.74; 1.12)	0.361			
Ever married	1.02	(0.66; 1.59)	0.924			
Education (ref: up to high school)						
University	0.87	(0.7; 1.09)	0.227			
Nationality (ref: Emirati)						
Arabic	1.92	(1.56; 2.38)	<0.001	1.62	(1.22; 2.17)	0.001
Western	2.13	(1.18; 3.86)	0.012	2.06	(1.08; 3.93)	0.029
Asian	1.83	(1.36; 2.45)	<0.001	1.7	(1.15; 2.5)	0.007
Other	1.92	(0.8; 4.64)	0.145	1.48	(0.6; 3.69)	0.396
Type of living (ref: house with a garden or yard)						
An apartment/house with no garden or yard	1.94	(1.6; 2.35)	<0.001	1.51	(1.15; 1.98)	0.003
Working Status (ref: Not working)						
Working from home	0.83	(0.67; 1.04)	0.595			
Working	0.89	(0.67; 1.18)	0.133			
Number of Children (ref: 0)						
1–2	1.14	(0.91; 1.43)	0.248	1.12	(0.88; 1.43)	0.349
≥3	0.75	(0.6; 0.95)	0.017	0.95	(0.71; 1.26)	0.708
BMI (kg/m^2^) (ref: <25)						
≥25	1.77	(1.43; 2.18)	<0.001	2.25	(1.79; 2.83)	<0.001

Abbreviations: BMI: Body Mass Index, Coef.: Coefficient, adj coef: adjusted coefficient, CI: Confidence interval. Model 1: Crude associations; Model 2: Adjusted for sex, age, nationality, type of living, number of children and BMI.

**Table A3:** Simple and multiple logistic regressions describing the association between decreased physical activity and sociodemographic characteristics in the study population (*n* = 2060).

	Decreased Physical Activity					
	Model 1			Model 2		
	Crude OR	95% CI	*p*-Value	Adj OR	95% CI	*p*-Value
Sex (ref: males)						
Female	1.25	(1; 1.56)	0.05	1.12	(0.88; 1.43)	0.354
Age (ref 18–30)						
31–40	1.13	(0.9; 1.42)	0.297	1.24	(0.98; 1.59)	0.081
>40	1.34	(1.05; 1.7)	0.018	1.61	(1.25; 2.08)	<0.001
Marital status (ref: Single)						
Married	1.01	(0.82; 1.24)	0.921			
Ever married	0.84	(0.53; 1.33)	0.448			
Education (ref: up to high school)						
University	0.76	(0.62; 0.95)	0.014	0.84	(0.67; 1.05)	0.125
Nationality (ref: Emirati)						
Arabic	0.61	(0.49; 0.76)	<0.001	0.7	(0.53; 0.93)	0.015
Western	0.62	(0.32; 1.2)	0.156	0.73	(0.36; 1.46)	0.367
Asian	0.76	(0.56; 1.03)	0.074	0.79	(0.54; 1.15)	0.213
Other	0.72	(0.28; 1.84)	0.488	0.74	(0.28; 1.96)	0.545
Type of living (ref: House with a garden or yard)						
An apartment/house with no garden or yard	1.50	(1.22; 1.81)	<0.001	1.25	(0.94; 1.66)	0.121
Working Status (ref: Not working)						
Working from home	0.72	(0.58; 0.9)	0.001	0.7	(0.55; 0.89)	0.003
Working	1.1	(0.84; 1.44)	0.496	1.06	(0.8; 1.42)	0.673
Number of Children (ref: 0)						
1–2	0.85	(0.68; 1.08)	0.184			
≥3	1.08	(0.86; 1.35)	0.517			
BMI (kg/m^2^) (ref: <25)						
>or = 25	1.32	(1.08; 1.61)	0.006	1.38	(1.13; 1.72)	0.002

Abbreviations: BMI: Body Mass Index, Coef.: Coefficient, adj coef: adjusted coefficient, CI: Confidence interval. Model 1: Crude associations; Model 2: Adjusted for sex, age, education, nationality, type of living, working status and BMI.

**Table A4:** Simple and multiple logistic regressions describing the association between decreased sleep and sociodemographic characteristics in the study population (*n* = 2060).

	Decreased Sleep					
	Model 1			Model 2		
	Crude OR	95% CI	*p*-Value	Adj OR	95% CI	*p*-Value
Sex (ref: males)						
Female	1.69	(1.29; 2.21)	<0.001	1.61	(1.22; 2.12)	<0.001
Age (ref 18–30)						
31–40	0.8	(0.63; 1.03)	0.081	0.85	(0.66; 1.1)	0.108
>40	0.66	(0.5; 0.86)	0.003	0.71	(0.54; 0.94)	0.003
Marital status (ref: Single)						
Married	0.89	(0.71; 1.12)	0.309			
Ever married	0.81	(0.48; 1.37)	0.439			
Education (ref: up to high school)						
University	0.92	(0.72; 1.17)	0.495			
Nationality (ref: Emirati)						
Arabic	0.89	(0.71; 1.13)	0.353	0.99	(0.77; 1.26)	0.913
Western	0.58	(0.26; 1.32)	0.194	0.68	(0.3; 1.55)	0.415
Asian	0.69	(0.48; 0.99)	0.042	0.74	(0.51; 1.07)	0.205
Other	1.63	(0.66; 4.05)	0.29	1.56	(0.62; 3.92)	0.395
Type of living (ref: House with a garden or yard)						
An apartment/house with no garden or yard	0.95	(0.76; 1.18)	0.508			
Working Status (ref: Not working)						
Working from home	1.16	(0.9; 1.5)	0.248			
Working	0.87	(0.63; 1.22)	0.423			
Number of Children (ref: 0)						
1–2	1.05	(0.81; 1.36)	0.733			
≥3	1.14	(0.89; 1.48)	0.298			
BMI (kg/m^2^) (ref: <25)						
>or = 25	1.22	(0.97; 1.53)	0.083			

Abbreviations: BMI: Body Mass Index, Coef.: Coefficient, adj coef: adjusted coefficient, CI: Confidence interval. Model 1: Crude associations; Model 2: Adjusted for sex, age and nationality.

**Table A5:** Simple and multiple logistic regressions describing the association between increased smoking and sociodemographic characteristics in the study population (*n* = 2060).

	Increased Smoking					
	Model 1			Model 2		
	Crude OR	95% CI	*p*-Value	Adj OR	95% CI	*p*-Value
**Sex (ref: males)**						
Female	**0.25**	**(0.14; 0.44)**	**<0.001**	**0.27**	**(0.15; 0.49)**	**<0.001**
**Age (ref 18–30)**						
31–40	0.78	(0.42; 1.46)	0.438	0.6	(0.31; 1.15)	0.126
>40	**0.45**	**(0.2; 0.99)**	**0.046**	**0.25**	**(0.11; 0.57)**	**0.001**
**Marital status (ref: Single)**						
Married	0.7	(0.39; 1.24)	0.222			
Ever married	0.29	(0.04; 2.22)	0.236			
**Education (ref: up to high school)**						
University	1.45	(0.7; 3)	0.319			
**Nationality (ref: Emirati)**						
Arabic	**4.06**	**(2.07; 7.96)**	**<0.001**	2.25	(0.95; 5.37)	0.066
Western	**5.61**	**(1.53; 20.56)**	**0.009**	4.15	(0.98; 17.56)	0.054
Asian	0.68	(0.15; 3.04)	0.611	0.33	(0.06; 1.69)	0.183
Other	**8.6**	**(1.81; 40.97)**	**0.007**	5.17	(0.99; 26.9)	0.051
**Type of living (ref: House with a garden or yard)**						
An apartment/house with no garden or yard	**2.82**	**(1.57; 5.05)**	**<0.001**	1.84	(0.86; 3.94)	0.119
**Working Status (ref: Not working)**						
Working from home	0.97	(0.47; 2.01)	0.938			
Working	1.92	(0.88; 4.18	0.102			
**Number of Children (ref: 0)**						
1–2	1.81	(0.98; 3.36)	0.058			
≥3	0.47	(0.19; 1.12)	0.089			
**BMI (kg/m^2^) (ref: <25)**						
>or = 25	**1.97**	**(1; 3.86)**	**0.05**	1.9	(0.93; 3.88)	0.077

Abbreviations: BMI: Body Mass Index, Coef.: Coefficient, adj coef: adjusted coefficient, CI: Confidence interval. Model 1: Crude associations; Model 2: Adjusted for sex, age, nationality, type of living and BMI. Numbers in bold are statistically significant (*p*-value < 0.05).
