# Supplementary material for: Indirect Health Effects of COVID-19: Unhealthy Lifestyle Behaviors during the Lockdown in the United Arab Emirates
Source: Int J Environ Res Public Health. 2021 Feb 18;18(4):1964. doi: 10.3390/ijerph18041964 (PMC7922937; doi:10.3390/ijerph18041964)
Supplement: Supplementary file 1 [file ijerph-18-01964-s001.pdf]

Nutrition and Lifestyle Changes During COVID 19 Lockdown in the UAE

Introduction

Dear Sir/Madam

It is our pleasure to invite you to participate in this online study. Your help in responding to this survey will help us learn more about changes in your dietary and lifestyle habits during the Corona lockdown which will help us in understanding the influence of the corona pandemic on the Nutritional Status of the population This study is conducted by Health Promotion in the Supreme Council for Family Affairs in Sharjah with the collaboration of the University of Sharjah. We appreciate your time and assure you confidentiality. The data will be kept strictly confidential and for scientific research purposes only. Your participation in this study is completely voluntary. You have the right to refuse or do not complete the questionnaire at any time and for any reason without any penalty or consequences. The survey should take approximately 10 min to complete.

If you have any questions regarding this study, please call or write to principal investigators Mrs Mahra A Kitbi from Health Promotion in the Supreme Council for Family Affairs in Sharjah (mahra@scfa.shj.ae telephone: 0506333089 and Dr. Hadia Radwan from the University of Sharjah (hradwan@sharjah.ac.ae) telephone: 065057549.

If you have concerns or complaints about this study, please write to the University of Sharjah Research Ethics Committee at rec@sharjah.ac.ae

Thank you for your interest in participating.

I have read all the information and agree to participate

*

/Agree

Your Health and Physical Activity

1. Quarantine is a good way to protect my family and to stop the spread of infectious disease outbreaks. *

Agree

Disagree

2. Are you living in: *

An apartment/house with no garden

A house with a garden or yard

3. Are you working? *

Yes

No

4. If yes, are you working from home?

Yes

No

5. Since lock down, did your physical activity ____? *

Increase
Decrease
Same

Dietary

6. Since lock down, did your food intake_______? *
Increase
Decrease
Same

7. Which food items do you consume the most during COVID-19 pandemic? (select all that applies) *
Fruits
Vegetables
Home Cooked food
Delivery food
Fried foods
Cakes and pastries
Chips and crackers
Fast food
Nuts
Sweets
Herbal Tea
None

8. What type of food you are consuming more *

Salty Snacks: chips, popcorn, crackers, etc
Sweet snacks: Cupcakes, cookies, cakes, etc
Cooked food
Nothing
Other

Lifestyle

9. Did your smoking during the COVID-19 pandemic --------? *

Decrease
Increase
It did not change
I am not a smoker

10. In general, how many times you used to go grocery shopping per week before COVID-19 lockdown? *

1 time/week
2 times/week
3 times or more/week
No definite time

11. In general, how many times you used to go grocery shopping per week during COVID-19 lockdown? *

1 time/week
2 times/week
3 times or more/week
No definite time

12. When you are going out, do you wear? *

Gloves
Mask
Both
None

13. How do you disinfect purchased fruits and vegetables? *

Water
Water + vinegar
Water + salt
Water + vinegar + salt
Water + Detergents (Dishwashing liquid)
Disinfectants for vegetables and fruits
None

14. Do you order food or groceries for delivery? *
Yes
No

15. Do you sterilize the food items when delivered? *

Yes
No

16. Did your hours of sleep at night ------------during lockdown?

Increase
Decrease
Same

17. Do you feel angry nowadays more than before? *

Yes
No

18. Are you feeling more stressed now during lockdown? *

Yes
No

19. Mainly, what do you do to relieve your stress? *

Eat more
Meditate
Pray
Watch movies/listen to music/social media
Exercise more
Others: _____

20. Do you think after lockdown your lifestyle will.......? *

Improve
Don't know if it will change
Stays the same

Information About You

21. Age *: ____________

22. Gender *

Male
Female
23. Marital status *

Married
Single
Divorced
Widowed

24. What is your Nationality?

Emirati
Arabic
Western
Asian
Other: __________

25. Education *

High school diploma or less
University degree

26. Residence at---------- *

Sharjah
Dubai
Ajman
UAQ
RAK
Fujairah
AbuDhabi

27. How many Children you have? ___________

28. Did your weight __________ during lockdown? *

Increase
Decrease
Did not change

29. Your Weight(cm): *

30. Your Height(kg): *

..... Thank You
